# Bewertung der Einsätze des Robert Koch-Instituts für COVID-19-Ausbruchsuntersuchungen durch subnationale Gesundheitsbehörden

**DOI:** 10.1007/s00103-025-04032-6

**Published:** 2025-03-19

**Authors:** Mario Martín-Sánchez, Claudia Siffczyk, Anna Loenenbach, Katja Kajikhina, Nadine Zeitlmann

**Affiliations:** 1 Abteilung für Infektionsepidemiologie, Robert Koch-Institut, Seestr. 10, 13353 Berlin, Deutschland; 2 ECDC Fellowship Programme, Field Epidemiology path (EPIET), European Centre for Disease Prevention and Control (ECDC), Stockholm, Schweden

**Keywords:** Ausbruchsuntersuchung, Feldteams, Krankheitsausbrüche, Krisenmanagement, COVID-19, Outbreak investigation, Task force, Disease outbreaks, Crisis management, COVID-19

## Abstract

**Hintergrund:**

Gemäß Infektionsschutzgesetz kann das Robert Koch-Institut (RKI) auf Amtshilfeersuchen (AHE) die Gesundheitsämter (GÄ) und Landesbehörden (LB) bei Ausbruchsuntersuchungen unterstützen. Während der COVID-19-Pandemie unterstützten RKI-Teams GÄ und LB bei ca. 50 Ausbruchsuntersuchungen (vor Ort oder virtuell). Gründe für oder gegen ein AHE sowie die Einschätzung stattgefundener RKI-Einsätze sollen erhoben und analysiert werden.

**Methoden:**

Im Zeitraum 09.03.2023–12.05.2023 stellte das RKI allen GÄ (*n* = 376) und LB (*n* = 16) den Link zu einer Online-Befragung zur Verfügung. Diese enthielt Fragen dazu, ob, wie oft und warum bzw. warum nicht ein AHE für ein RKI-Team gestellt wurde. Wenn ein Einsatz stattfand, wurde um eine Bewertung hinsichtlich Nützlichkeit, Rechtzeitigkeit, Zufriedenheit und des Arbeitsaufwandes gebeten.

**Ergebnisse:**

146 Behörden (136 GÄ und 10 LB) nahmen teil; 21 (14 %) stellten mindestens ein AHE und gaben Feedback zu 22 Einsätzen vor Ort. Häufige Gründe für das Nichtstellen waren kein Bedarf (60 %, 56/94) und keine Erfahrungen mit (31 %, 29/34) oder Kenntnis zu AHE-Prozessen (29 %, 27/94). Die 22 Einsätze fanden zwischen Februar 2020 und September 2021 statt. Gründe für Unterstützungsanfragen waren fehlende fachliche Expertise (*n* = 18), personelle Ressourcen (*n* = 13) oder politischer/öffentlicher Druck (*n* = 12). Größter Nutzen der Einsätze war die Beantwortung epidemiologischer Fragestellungen (*n* = 18). Bei 20 Einsätzen waren die Behörden mit dem Einsatz zufrieden; bei 19 war das RKI-Team rechtzeitig vor Ort. Die Mehrarbeit für die amtshilfeersuchenden Behörden wurde als angemessen bewertet.

**Diskussion:**

GÄ und LB bewerteten die RKI-Einsätze positiv. Bezüglich der AHE-Prozesse zeigte sich Informationsbedarf, der Schulungen und Bereitstellung von Informationsmaterial durch das RKI einschloss.

**Zusatzmaterial online:**

Zusätzliche Informationen sind in der Online-Version dieses Artikels (10.1007/s00103-025-04032-6) enthalten.

## Abstract (en)

**Background:**

Under the German Infection Protection Act, the Robert Koch Institute (RKI) can support local health authorities (LHAs) and state health authorities (SHAs) in outbreak investigations after an administrative request for assistance. During the COVID-19 pandemic, about 50 RKI teams supported LHAs and SHAs in outbreak investigations (virtually or on site). Reasons for or against requesting assistance as well as the assessment of outbreak deployments that have taken place should be collected and analysed.

**Methods:**

Between 9 March 2023 and 12 May 2023, the RKI provided all LHAs (*n* = 376) and SHAs (*n* = 10) with a link to an online survey that included questions about whether, how often, and why or why not an RKI field team was requested. If a deployment took place, we asked for an assessment of the usefulness and timeliness of the deployment, their satisfaction with it and the work load involved.

**Results:**

A total of 146 authorities (136 LHAs and 10 SHAs) responded; 21 (14%) requested an RKI team at least once and gave feedback on 22 deployments. Common reasons for not requesting were no need (60%, 56/94) and no experience with (31%, 29/34) or knowledge of the assistance request process (29%, 27/94). The 22 deployments took place between February 2020 and September 2021. The reasons for requesting assistance were lack of professional expertise (*n* = 18), personnel resources (*n* = 13) or political/public pressure (*n* = 12). The biggest benefit of the RKI deployments was answering epidemiological questions (*n* = 18). The SHAs and LHAs were satisfied with 20 of the deployments and the RKI team arrived on time for 19. The additional workload for the requesting authorities was judged to be reasonable.

**Discussion:**

LHAs and SHAs rated the RKI deployments positively. Regarding the assistance request processes, there was a need for information that should be covered by training and provision of information material.

## Hintergrund

Am 27.01.2020 wurde die erste Coronavirus-19-Erkrankung (COVID-19) in Deutschland laborbestätigt [1]. Rund 6 Wochen später, am 11.03.2020 erklärte die Weltgesundheitsorganisation (WHO) den Ausbruch von COVID-19 zur globalen Pandemie und auch der Deutsche Bundestag stufte wenig später, am 25.03.2020, die Verbreitung von COVID-19 als eine Epidemie von nationaler Bedeutung ein [2, 3].

Von Beginn bis Ende der Pandemie am 05.05.2023 wurden in Deutschland ca. 38,4 Mio. COVID-19-Fälle gezählt [4]. COVID-19-Ausbrüche hatten in Deutschland je nach Pandemiephase unterschiedlichen Charakter. Zu Beginn, in den ersten 2 Monaten des Jahres 2020, traten vor allem sporadische und importierte Fälle sowie kleinere, regional begrenzte Ausbrüche auf. Von März bis Mai 2020, während der ersten COVID-19-Welle in Deutschland, kam es vermehrt zu Ausbrüchen in Krankenhäusern sowie Alten- und Pflegeheimen. In den darauffolgenden Sommermonaten 2020 wurden vor allem größere überregionale Ausbrüche in Betrieben beobachtet und verschiedene Orte für Freizeitaktivitäten als wichtige Ausbruchssettings identifiziert [5]. Bis Mitte August 2020 wurden 55.141 (27 %) der 202.225 gemeldeten COVID-19-Fälle mindestens einem Ausbruch zugeordnet. Von diesen 55.141 Fällen lagen bei 50.755 (92 %) Angaben zum Infektionsumfeld vor. Von diesen entfallen 13.314 (26 %) auf Alten- und Pflegeheime und 4107 (8 %) auf Krankenhäuser. In Alten- und Pflegeheimen wurde der höchste Fall-Verstorbenen-Anteil beobachtet (19 %; [6]). Die Untersuchung und Kontrolle von COVID-19-Ausbrüchen in diesem Setting war von entscheidender Bedeutung, da dort Hochrisikogruppen (ältere Menschen, immungeschwächte Personen und Personen mit Komorbiditäten) betreut wurden, deren Erkrankungs- und Sterblichkeitsrisiko generell, aber auch ggf. durch einrichtungsspezifische Bedingungen erhöht gewesen sein konnte [7]. Im September 2020 begann eine zweite Erkrankungswelle, die von deutlich höheren Fallzahlen geprägt war. Ausbrüche in privaten Haushalten und am Arbeitsplatz traten in jeder Phase der Pandemie auf [5]. Insgesamt wurden im Jahr 2020 von 1.785.656 COVID-19-Fällen, die der Referenzdefinition entsprachen, 22 % (*n* = 385.730) als Teil eines Ausbruchs übermittelt [8].

Gemäß Infektionsschutzgesetz (IfSG) obliegt es in Deutschland den Gesundheitsämtern (GÄ), die erforderlichen Maßnahmen zur Prävention und Eindämmung übertragbarer Krankheiten durchzuführen. Dies schließt auch die Durchführung von Ausbruchsuntersuchungen ein [9, 10], welche z. B. zum Ziel haben, Infektionsketten bzw. -vehikel zu identifizieren, Infektionsketten zu unterbrechen und die Anzahl der Erkrankten so gering wie möglich zu halten. Während der COVID-19-Pandemie boten Ausbruchsuntersuchungen zusätzlich die Gelegenheit, das Infektionsgeschehen und die Eigenschaften des neuartigen Erregers SARS-CoV‑2 in Deutschland besser zu verstehen [11].

Bei Bedarf kann das Robert Koch-Institut (RKI) als nationale Einrichtung des Bundes auf dem Gebiet der Krankheitsüberwachung und -prävention auf Anfrage GÄ oder Landesbehörden (LB) bei der Überwachung, Prävention und Bekämpfung schwerwiegender übertragbarer Krankheiten (z. B. im Rahmen von Ausbruchsuntersuchungen) unterstützen [10]. Um diese Unterstützung zu beantragen und dem RKI die Einsicht in personenbezogene Daten zu ermöglichen, erfolgt das Amtshilfeersuchen gemäß § 4 IfSG auf Anfrage der obersten LB an das RKI.

Während der COVID-19-Pandemie konnte eine Unterstützung durch das RKI durch Amtshilfe auf verschiedene Weise erfolgen: 1) bei Ausbruchsuntersuchungen durch die Entsendung bzw. Zusammenstellung eines RKI-Ausbruchsteams (RKI-Einsätze), 2) durch die Entsendung von „Containment Scouts“ des RKI zur Verstärkung der Kapazitäten für v. a. Kontaktpersonennachverfolgung und -management [11, 12]. Bei Ersterem konnte strukturierte fachlich epidemiologische oder strategische Unterstützung vor Ort (RKI-Feldeinsätze) oder in virtueller bzw. telefonischer Form (RKI-Ferneinsätze) geleistet werden.

Zwischen Januar 2020 und September 2021 fanden 47 RKI-Einsätze zur Unterstützung bei COVID-19-Ausbruchsuntersuchungen statt, davon waren 30 RKI-Feldeinsätze und 17 RKI-Ferneinsätze. Der erste COVID-19-Feldeinsatz begann am 30.01.2020 [11].

Im Juni 2022 fand am RKI eine Feedbackrunde unter den Mitarbeitenden der RKI-Ausbruchsteams statt. Die Teilnehmenden berichteten über ihre vielfältigen Erfahrungen während der Einsätze und identifizierten offene Fragen, für deren Beantwortung eine umfangreichere Betrachtung und Bewertung des Nutzens dieser RKI-Einsätze notwendig erschien. Diese sollte die noch fehlende Perspektive der amtshilfeersuchenden Behörden (GÄ und LB) einschließen.

Die hier vorgestellte Studie verfolgte demnach 2 wichtige Zielstellungen: Zum einen sollte durch die Befragung von GÄ und LB untersucht werden, wie oft und aus welchen Gründen Amtshilfeersuchen für RKI-Einsätze zur Untersuchung von COVID-19-Ausbrüchen in Anspruch genommen oder unterlassen wurden. Zweitens sollten die aus den Amtshilfeersuchen resultierten RKI-Einsätze durch Rückmeldungen der entsprechenden Behörden hinsichtlich ihrer Nützlichkeit, Rechtzeitigkeit, Zufriedenheit und Arbeitsbelastung für GÄ und LB bewertet werden.

Die Studienergebnisse sollen dabei helfen, Aspekte der RKI-Einsätze zu identifizieren, die gut funktioniert haben und im Rahmen der Koordination zukünftiger RKI-Ausbruchsteams fortgesetzt werden sollten. Zudem sollen mögliche Hindernisse für das Stellen eines Amtshilfeersuchens aufgedeckt und Verbesserungsvorschläge für zukünftige RKI-Einsätze zusammengetragen werden.

## Methoden

### Studiendesign.

Zwischen dem 09.03.2023 und dem 12.05.2023 führte das RKI eine Querschnittserhebung (Online-Befragung) unter allen GÄ (*n* = 376) und LB (*n* = 16) in Deutschland durch, welche jeweils eine Antwort pro Behörde vorsah.

### Fragebogen.

Der Befragungsinhalt wurde auf der Grundlage der Ergebnisse der zuvor beschriebenen Feedbackrunde zusammengestellt. Der Fragebogen (siehe Onlinematerial 1) umfasste 4 offene Fragen (Freitextantworten) und 23 geschlossene Fragen (u. a. Likert-Skalen). Viele geschlossene Fragen enthielten Unterfragen und die Möglichkeit, in der Auswahlkategorie „Sonstiges“ weitere Informationen anzugeben, wenn diese in der Hauptfrage nicht vorgesehen waren.

Der Fragebogen enthielt einen Abschnitt, der alle GÄ und LB adressierte, sowie spezifische Abschnitte nur für diejenigen Behörden, die ein Amtshilfeersuchen gestellt hatten. Darunter waren auch Abschnitte nur für Behörden, die zwischen Januar 2020 und September 2021 mindestens einen RKI-Einsatz für eine Ausbruchsuntersuchung hatten. Der Abschnitt zu den RKI-Einsätzen konnte von den Behörden für jeden Einsatz separat ausgefüllt werden, je nachdem, wie viele verschiedene Einsätze von RKI-Ausbruchsteams in der Behörde durchgeführt wurden. Der Fragebogen wurde den teilnehmenden Behörden mithilfe der Befragungsplattform „Voxco“ (Acuity 4 Survey, Voxco, Montreal, Canada) online zur Verfügung gestellt, nachdem dieser vorab inhaltlich und bezüglich seiner technischen Umsetzung intern im RKI und mit Mitarbeitenden von LB und GÄ pilotiert und angepasst wurde.

### Rekrutierung.

Zur Rekrutierung der Teilnehmenden wurde der Link zur Online-Befragung mehrfach sowohl über die wöchentliche Epidemiologische Lagekonferenz (EpiLag) des RKI, der 16 Landesbehörden und der Bundeswehr [13, 14] als auch über „Agora“, eine Kollaborationsplattform für den Öffentlichen Gesundheitsdienst (ÖGD), verteilt [15]. Zusätzlich wurde die Befragung über das Netzwerk der ÖGD-Feedbackgruppe und das Forschungs‑, Trainings- und Evidenznetzwerk für die Öffentliche Gesundheit (ÖGD-FORTE) beworben. Die Adressaten der Befragung wurden angewiesen, die Befragung nur einmal pro Behörde auszufüllen. Die Beantwortung sollte durch die jeweiligen Amtsleitungen bzw. Bereichsleitungen gegebenenfalls unter Hinzuziehung von oder durch Weiterleitung an Mitarbeitende erfolgen, die an den jeweiligen durch das RKI unterstützten Ausbruchsuntersuchungen beteiligt waren.

### Datenanalyse.

Einschlusskriterium einer Fragebogenantwort in die Studie war die Antwort auf eine Schlüsselfrage (Frage Nr. 5, s. Onlinematerial 1): „Wurde das RKI von Ihrer Behörde im Zeitraum Januar 2020 – September 2021 bzgl. COVID-19 um Unterstützung durch ein Amtshilfeersuchen gebeten?“ Diese Frage wurde auf der Grundlage der Ziele der Studie als Schlüsselfrage ausgewählt sowie einer Voranalyse der Daten, die darauf hindeutete, dass einige Behörden die ersten Fragen des Fragebogens (vor der Schlüsselfrage) möglicherweise mehrfach beantwortet haben.

Um die Antwortquoten zu berechnen, haben wir die Anzahl der teilnehmenden Behörden, wie oben definiert, als Zähler und die Gesamtanzahl der oberen LB (*n* = 16) und der GÄ in Deutschland (*n* = 376) mit Stand 27.04.2022 als Nenner verwendet [16]. Alle deskriptiven Datenanalysen wurden mit R Statistical Software (v4.1.2; [17]) oder Excel durchgeführt. Als Analyseeinheiten fungierten für die allgemeinen Abschnitte sowie die Abschnitte zu Amtshilfeersuchen die teilnehmenden Behörden, für die Abschnitte zu den Einsätzen der RKI-Ausbruchsteams die Einsätze. Bei kategorischen Fragen und Likert-Skalen analysierten wir die Anzahl der Antworten und den prozentualen Anteil dieser an der Gesamtzahl. Bei quantitativen Variablen berichteten wir den Median und den Interquartilsabstand (IQR). Freitextantworten wurden inhaltsanalytisch mit induktiver Kategorienbildung ausgewertet.

### Ethik und Datenschutz.

Die durch die Befragung erhobenen Daten wurden anonym (nach einer schriftlichen Einverständniserklärung zur Teilnahme) erhoben und enthielten weder Personen- noch Behördenbezug. Teilnehmende wurden zudem im Einleitungstext der Befragung angewiesen auch in Freitextantworten von Nennungen abzusehen, die eine Behördenzugehörigkeit oder einen Rückschluss auf das Individuum, das den Fragebogen ausfüllte, nahelegen könnten.

Da die Bewertung der RKI-Einsätze im Rahmen der Routinetätigkeiten des Ausbruchskoordinationsteams der ÖGD-Kontaktstelle am RKI stattfand und keine sensiblen, gesundheitsbezogenen und personenbezogenen Daten erhoben wurden, wurde von einem Ethikvotum für die Studie abgesehen. Eine schriftliche Datenschutzfolgeabschätzung wurde jedoch vorab der RKI-Datenschutzabteilung vorgelegt und im Januar 2023 genehmigt.

## Ergebnisse

### Beschreibung der Teilnehmenden (GÄ und LB)

**Abb. 1:**
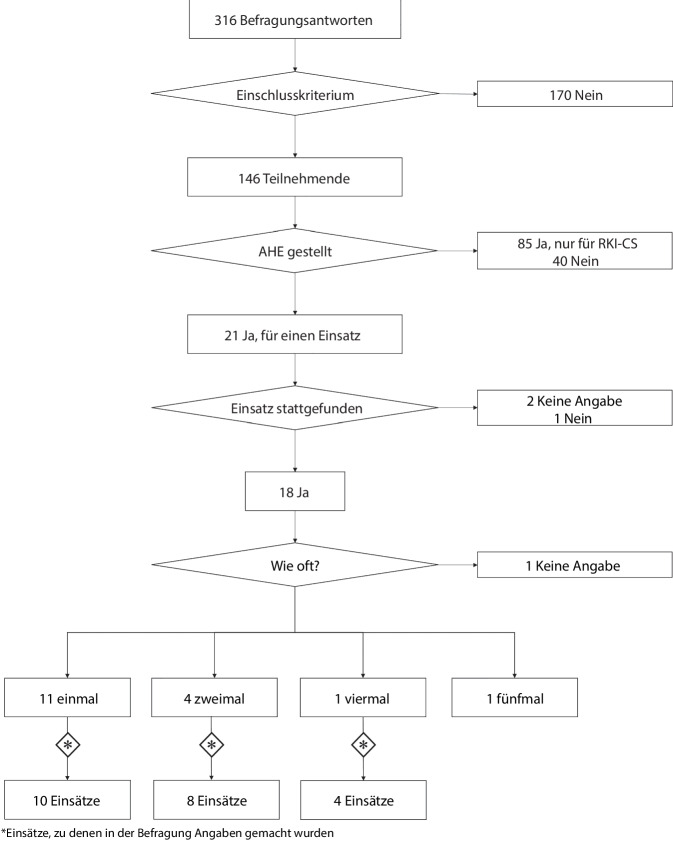
Flussdiagramm zur Ermittlung der Befragungsteilnehmenden und der endgültigen Analyseeinheiten (Gesundheitsbehörden und Einsätze) für die Bewertung der RKI-Einsätze zur Unterstützung der subnationalen Gesundheitsbehörden in Deutschland während der COVID-19-Pandemie, 2020–2021. *Quelle*: eigene Abbildung. *AHE* Amtshilfeersuchen, *RKI-CS* „Containment Scouts“ des Robert Koch-Instituts

Aus insgesamt 316 Befragungsantworten klassifizierten wir 146 Behörden (anhand des oben genannten Einschlusskriteriums) als „teilnehmende Behörden“ in unserer Befragung (Abb. 1). Unter diesen befanden sich 136 GÄ und 10 LB. Dies entspricht einer Antwortquote von 36 % für die GÄ (136/376) und 63 % für die LB (10/16). Bei den teilnehmenden GÄ handelte es sich in den meisten Fällen um Behörden aus Landkreisen mit über 100.000 Einwohnenden (Tab. 1).

**Tab. 1:** Merkmale der teilnehmenden Gesundheitsämter (GÄ) und oberen Landesbehörden (LB)

		GÄ (*N* = 136)		LB (*N* = 10)		Insgesamt (*N* = 146)	
		*n*	*%*	*n*	*%*	*n*	*%*
Größe des zugehörigen Kreises (Anzahl Einwohnende)	< 100.000	27	20	–	–	–	–
	100.000–200.000	56	41	–	–	–	–
	200.001–300.000	23	17	–	–	–	–
	300.001–400.000	12	9	–	–	–	–
	400.001–500.000	7	5	–	–	–	–
	> 500.000	10	7	–	–	–	–
	Keine Angabe	1	1	–	–	–	–
Größe des zuständigen Fachbereiches (Anzahl Mitarbeitende)	1–10	68	50	2	20	70	48
	11–20	24	18	2	20	26	18
	21–30	9	7	0	0	9	6
	> 30	17	13	0	0	17	12
	Keine Angabe	18	13	6	60	24	16
Zusätzlich rekrutiertes Personal für COVID-19, zeitgleich arbeitend (Anzahl Mitarbeitende)	Keine	3	2	0	0	3	2
	1–10	3	2	4	40	7	5
	11–100	86	63	5	50	91	62
	> 100	41	30	0	0	41	28
	Weiß nicht	2	2	1	10	3	2
	Keine Angabe	1	1	0	0	1	1
Training/Erfahrung zu Ausbruchsuntersuchungen*	Ausbildung zu:r Hygienekontrolleur:in/Gesundheitsaufseher:in	127	96	3	30	130	91
	Fachärztliche Ausbildung für ÖGW	84	63	3	30	87	61
	Langjährige praktische Erfahrungen mit Ausbruchsuntersuchungen	75	56	7	70	82	57
	Epikurs	14	11	4	40	18	13
	Postgraduiertenausbildung für angewandte Epidemiologie (FETP)	2	2	5	50	7	5
Erstellung eines Amtshilfeersuchens	Ja, für den Einsatz eines RKI-Feldteams	16	12	5	50	21	14
	Nein	37	27	3	30	40	27
	Ja, aber ausschließlich zur Anfrage von RKI-CS	83	61	2	20	85	58

*ÖGW* Öffentliches Gesundheitswesen, *RKI-CS* Containment Scouts des Robert Koch-Instituts

*Mehrfachantworten möglich, Anzahl und Anteil der positiven Antworten an der Gesamtzahl ohne 3 Gesundheitsämter, von denen keine Antworten zum Fragenblock gegeben wurden (Nenner: GÄ = 133, insgesamt = 143)

Die GÄ und LB sollten zudem die Anzahl der Mitarbeitenden im Fachbereich für Infektionsschutz und Ausbruchsuntersuchung der Behörde im Zeitraum vor der Pandemie angeben sowie die maximale Anzahl der während der Pandemie zusätzlich rekrutierten Mitarbeitenden für die COVID-19-Arbeit. Diese maximale Anzahl lag bei den GÄ meist zwischen 11 und 100 Personen (63 %, 86/136) und bei den LB meist zwischen 1–10 (40 %, 4/10) und 11–100 Personen (50 %, 5/10; Tab. 1).

Hinsichtlich der Aus- und Weiterbildung sowie der Arbeitserfahrungen im Bereich Ausbruchsuntersuchungen gab es zwischen den Mitarbeitenden der GÄ und LB Unterschiede: Während die Mitarbeitenden der GÄ für diese Bereiche vorwiegend Ausbildungsabschlüsse in den Bereichen Hygienekontrolle/Gesundheitsaufsicht (96 %, 127/133) sowie Weiterbildungen zu Facharzt/-ärztin für Öffentliches Gesundheitswesen (ÖGW) vorwiesen (63 %, 84/133), waren unter den Mitarbeitenden der LB Weiterbildungen wie der „Epikurs@RKI“ (40 %, 4/10) oder Postgraduiertenausbildungen in angewandter Epidemiologie (z. B. PAE oder EPIET) weiter verbreitet (50 %, 5/10; [18, 19]; Tab. 1).

### Amtshilfeersuchen (Art und Motivation)

Insgesamt 16 der an der Studie teilnehmenden GÄ (12 %, 16/136) und 5 LB (50 %, 5/10) stellten ein Amtshilfeersuchen für den Einsatz eines RKI-Ausbruchsteams während der COVID-19-Pandemie. Insgesamt 37 GÄ und 3 LB stellten kein Amtshilfeersuchen und 83 der GÄ und 2 der LB stellten ein Amtshilfeersuchen, jedoch ausschließlich für den Empfang von „Containment Scouts“ des RKI.

Von den 125 Behörden, die kein Amtshilfeersuchen oder nur ein Amtshilfeersuchen für „Containment Scouts“ des RKI gestellt haben, haben 94 (92 GÄ und 2 LB) weitere Informationen zur Verfügung gestellt. Der häufigste Grund für das Nichtstellen eines Amtshilfeersuchens für ein RKI-Ausbruchsteam war ein hierfür fehlender Bedarf (61 %; 57/94 der Beantwortenden). Unter den verbleibenden Antworten wurden mehrheitlich mangelnde Erfahrung mit den (31 %; 29/94) sowie Unklarheit bzw. Unkenntnis des Verfahrens zu Amtshilfeersuchen (29 %; 27/94) als Gründe genannt.

Ein Großteil erklärte, dass sie im zukünftigen Bedarfsfall wahrscheinlich (61 %, 57/94) oder sehr wahrscheinlich (4 %, 4/94) an dem Einsatz eines RKI-Ausbruchsteams interessiert wären. Diejenigen, die an einem Einsatz eines RKI-Ausbruchsteams eher kein Interesse hatten (35 %, 33/94), nannten im Freitext folgende Hauptgründe: Vorliegen von ausreichendem Fachwissen und Ressourcen in der eigenen Behörde (5 Behörden); zu hoher Aufwand oder Bürokratie des Prozesses (5 Behörden); mangelnde Erfahrung bzw. mangelndes Wissen über die Vorgehensweise bei der Stellung eines Amtshilfeersuchens (4 Behörden) sowie Unklarheit in der Zielstellung und der Aufgabenverteilung innerhalb eines Einsatzes (4 Behörden).

### Beschreibung der RKI-Einsätze

Von den 21 Behörden, die Amtshilfe für einen RKI-Einsatz ersucht hatten, gaben 18 an, dass mindestens ein Einsatz daraus resultierte, sowie eine Behörde, dass kein Einsatz nach dem Amtshilfeersuchen stattfand (Abb. 1). 2 Behörden machten keine Angaben.

15 Behörden (12 GÄ und 3 LB) gaben Auskunft zu insgesamt 22 RKI-Einsätzen während der Pandemie. In 10 Behörden erfolgte ein Einsatz, in 4 Behörden fanden jeweils 2 Einsätze statt und in einer Behörde wurden in 4 Einsätzen RKI-Ausbruchsteams empfangen (Abb. 1). Die 22 Einsätze fanden zwischen Februar 2020 und September 2021 vor Ort statt und hatten eine mediane Dauer von 4 Tagen (Interquartilsabstand, IQR: 2,5–6,5). Die am häufigsten genannten Ausbruchssettings, in denen ein Einsatz stattfand, waren Alten‑/Pflegeheime (12 Einsätze), gefolgt von Arbeitsplatz/Betrieb (5 Einsätze) und privater Haushalt (5 Einsätze; Tab. 2, siehe Onlinematerial 2 für Ergebnisse stratifiziert nach GÄ und LB).

**Tab. 2:** Angaben zu den Einsätzen des Robert Koch-Instituts (RKI) zur Unterstützung der subnationalen Gesundheitsbehörden in Deutschland während der COVID-19-Pandemie, 2020–2021

		Einsätze gesamt (*N* = 22)	
		*n*	*%*
*Variablen mit nur einer möglichen Antwort*			
**Beschreibung des Einsatzes**			
Monat des Beginns	Januar-Juni 2020	9	41
	Juli-Dezember 2020	2	9
	Januar-Juni 2021	9	41
	Juli-September 2021	2	9
Dauer des Einsatzes	≤ 5 Tage	14	64
	6–10 Tage	1	5
	> 10 Tage	4	18
	Keine Angabe	3	14
Größe des RKI-Feldteams	2 Personen	4	18
	3 Personen	6	27
	4 Personen	8	36
	5 Personen	1	5
	Keine Angabe	3	14
*Variablen mit Mehrfachantwortmöglichkeit*			
**Ausbruchssettings**^**a**^			
Alten‑/Pflegeheime	–	12	55
Arbeitsplatz/Betrieb	–	5	23
Privater Haushalt	–	5	23
Medizinische Einrichtung	–	4	18
Andere Ausbruchssettings	–	9	41
Zusätzliche fachliche Expertise benötigt	–	18	82
Fehlende personelle Ressourcen für Maßnahmen	–	13	59
Politischer und/oder öffentlicher Druck	–	12	55
Sonstige	–	1	5
**Einsatzvorbereitung**^**a**^			
Die Ziele des Einsatzes wurden im Voraus festgelegt	–	18	82
Die Erwartungen an das RKI-Team wurden festgelegt	–	18	82
Konkrete Ergebnisse/Outputs wurden vereinbart	–	12	60^**b**^
Eine Vorbesprechung mit dem RKI wurde durchgeführt	–	15	68

^**a**^ Mehrfachantworten möglich, Prozentangaben beziehen sich auf den Anteil der Ja-Antworten an der Gesamtzahl der Antworten innerhalb eines Fragenblocks

^b^ 2 „Weiß-nicht“-Antworten (Nenner = 20)

Die Behörden gaben verschiedene Motivationen für die Anfrage eines RKI-Ausbruchsteams an: In 18 Einsätzen wurde zusätzliche fachliche Expertise benötigt, in 13 Einsätzen wurden fehlende personelle Ressourcen für Maßnahmen angegeben sowie in 12 Einsätzen der politische und/oder öffentliche Druck als Grund genannt (Tab. 2).

Für 15 Einsätze gaben die Behörden an, dass eine Vorbesprechung mit dem RKI durchgeführt wurde; für 7 Einsätze wurde dies verneint.

Unabhängig davon wurde angegeben, dass bei der Mehrheit der Einsätze bei der Einsatzvorbereitung (*n* = 18) konkrete Ziele und Erwartungen an das RKI-Ausbruchsteam vorab festgelegt wurden. Bei 12 der 22 Einsätze wurden konkrete zu erwartende Ergebnisse/Outputs mit den RKI-Ausbruchsteams vereinbart, bei 8 Einsätzen war dies nicht der Fall (für 2 Einsätze keine Angabe; Tab. 2).

### Nützlichkeit, Rechtzeitigkeit und Zufriedenheit mit den RKI-Einsätzen

#### Nützlichkeit.

**Abb. 2:**
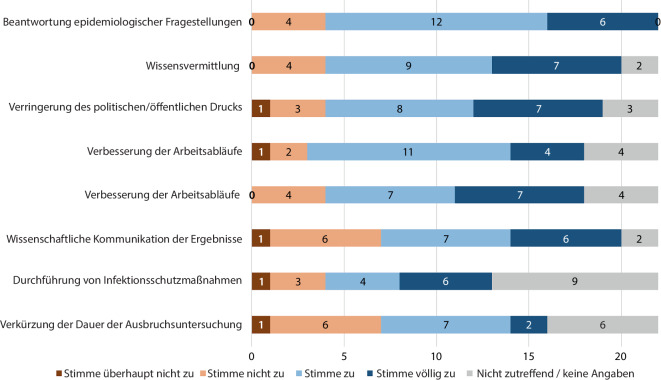
Bewertung des Nutzens der Einsätze des Robert Koch-Instituts (RKI) zur Unterstützung der subnationalen Gesundheitsbehörden in Deutschland während der COVID-19-Pandemie, 2020–2021. *Quelle*: eigene Abbildung

Die Mitarbeitenden des RKI haben die amtshilfeersuchenden Behörden bei den meisten Einsätzen wirksam unterstützt (14 von 16 Behörden mit auswertbaren Antworten für diese Frage beantworteten diese Aussage mit Zustimmung). Die häufigsten positiven Ergebnisse, die durch den RKI-Einsatz erzielt wurden, waren die Beantwortung epidemiologischer Fragen (18 Einsätze), die Vermittlung neuen Wissens (16 Einsätze) durch die RKI-Mitarbeitenden und die Verringerung des politischen und/oder öffentlichen Drucks für die Behörde (15 Einsätze; Abb. 2). Die Verkürzung der Dauer der Ausbruchsuntersuchung und die Durchführung von Infektionsschutzmaßnahmen durch den Einsatz des RKI-Ausbruchsteams wurden in jeweils 9 Einsätzen erreicht.

#### Rechtzeitigkeit und Einsatzdauer.

Das RKI war bei den meisten Einsätzen schnell genug vor Ort (19 von 22 Einsätzen). Bei 19 von 21 Einsätzen (1 Antwort nicht auswertbar) war die Einsatzdauer angemessen, bei 2 Einsätzen wurde sie als zu kurz angegeben. Bezüglich der Rechtzeitigkeit der Erstellung des Ausbruchsberichts durch das RKI wurde im Freitext für 3 Einsätze mitgeteilt, dass diese zu lange gedauert hätte oder dass ein schnellerer Abschluss der Berichtserstellung sinnvoll gewesen wäre.

#### Zufriedenheit.

Im Allgemeinen waren die amtshilfeersuchenden Behörden mit den Einsätzen der RKI-Ausbruchsteams zufrieden (7 Einsätze) oder sehr zufrieden (13 Einsätze). In diesem Zusammenhang waren die Behörden in 20 von 22 Einsätzen mit der Erreichbarkeit der Ansprechpersonen im RKI, in 19 von 22 Einsätzen mit der Kommunikation des RKI und in 20 von 21 Einsätzen (1 Einsatz lieferte keine Antwort auf diese Frage) mit den sonstigen Aspekten der Zusammenarbeit zufrieden.

### Arbeitsbelastung der amtshilfeersuchenden Behörden

Für 12 Einsätze stimmten die Behörden völlig zu oder zu, dass ihre Arbeitsbelastung durch den RKI-Einsatz verringert werden konnte. Bei 9 Einsätzen waren die Behörden nicht dieser Meinung.

**Abb. 3:**
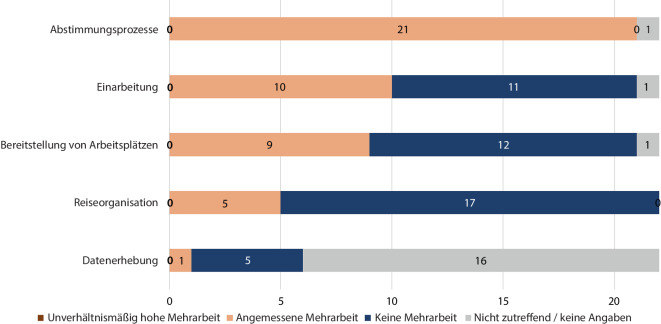
Bewertung der entstandenen Mehrarbeit in verschiedenen Arbeitsbereichen der Gesundheitsbehörden durch die Einsätze des Robert Koch-Instituts (RKI) zur Unterstützung der subnationalen Gesundheitsbehörden in Deutschland während der COVID-19-Pandemie, 2020–2021. *Quelle*: eigene Abbildung

Für die Reiseorganisation und die Bereitstellung von Arbeitsplätzen für die Mitglieder der RKI-Einsatzteams, deren Einarbeitung sowie für weitere Abstimmungsprozesse und Datenerhebung haben die Einsätze in einigen Fällen eine Mehrarbeit für die amtshilfeersuchenden Behörden verursacht. Dies gilt insbesondere für die Abstimmungsprozesse (bei allen 22 Einsätzen) und die Einarbeitung (bei 10 Einsätzen). Eine unverhältnismäßig hohe Mehrarbeit wurde jedoch bei keinem Einsatz angegeben (Abb. 3).

## Diskussion

In dieser Studie wollten wir den Prozess der Amtshilfeersuchen durch die Gesundheitsbehörden während der COVID-19-Pandemie bewerten und die vergangenen RKI-Einsätze, die im Rahmen des Amtshilfeersuchens in dieser Zeit durchgeführt wurden, evaluieren. Zusammenfassend haben insgesamt 136 GÄ und 10 LB an der Studie teilgenommen, von denen 21 (14 %) mindestens ein Amtshilfeersuchen für ein RKI-Einsatz gestellt haben. Als Gründe, warum kein Amtshilfeersuchen für ein RKI-Einsatz angefordert wurde, wurden neben „kein Bedarf“ häufig fehlende Erfahrungen und Kenntnisse über den Ablauf von Amtshilfeersuchen genannt. Wir erhielten Informationen aus 22 RKI-Feldeinsätzen, die zwischen Februar 2020 und September 2021 vor Ort stattfanden und zumeist im Setting Alten‑/Pflegeheim durchgeführt wurden. Fehlende fachliche Expertise und personelle Ressourcen sowie politischer/öffentlicher Druck wurden von den Behörden als Hauptgründe für die Anforderung eines RKI-Einsatzes genannt. Dazu passend zeigt unsere Studie, dass diese Hauptmotivationen sich auch für die meisten Behörden mit den größten Wirkungsfeldern der RKI-Einsätze decken: Am häufigsten wurden hier die Beantwortung epidemiologischer Fragen und die Verringerung des politischen/öffentlichen Drucks genannt. Bei 20 der 22 Einsätze waren die unterstützten Behörden mit dem Einsatz des RKI zufrieden, bei 19 Einsätzen waren Zeit und Dauer angemessen, einige Behörden gaben jedoch an, dass die Ausbruchsberichte möglichst zeitnah erstellt werden sollten. Auf der anderen Seite wurde jedoch angemerkt, dass die Einladung eines RKI-Ausbruchsteams auch zu einer Mehrarbeit in den amtshilfeersuchenden Behörden führen kann, insbesondere bei Abstimmungsprozessen. Dieser Mehraufwand war für die Behörden jedoch angemessen.

Ein wichtiges Ergebnis der Studie ist, dass der Prozess zur Stellung der Amtshilfe für ein Drittel der Antwortenden unklar oder unbekannt war. Dieses Verfahren wird in IfSG § 4 Abs. (1), Satz 5 und 6 beschrieben. Das RKI strebt aber an, dem ÖGD noch mehr Informationen über den Ablauf bzw. die Schritte des Amtshilfeersuchens und die möglichen Formen der Unterstützung zu geben. Im September 2020 wurde im RKI die „Kontaktstelle für den Öffentlichen Gesundheitsdienst“ eingerichtet [8] und im Jahr 2021 in diesem Rahmen auch ein Team für die Unterstützung von RKI-Einsätzen aufgebaut. Dieses Team befasst sich unter anderem mit der Koordination von Amtshilfeersuchen des ÖGD, der Zusammenstellung und Betreuung von Ausbruchsteams sowie der Zusammenstellung von Leitfäden und Arbeitshilfen. Zudem gehört es zu seinen Aufgaben, Vorträge zu Amtshilfeersuchen und RKI-Einsätzen in einschlägigen Gremien zu halten (z. B. ÖGD-Forum, gemeinsam mit der Akademie für Öffentliches Gesundheitswesen durchgeführtes Webseminar „Wissenschaft trifft Praxis“, Bund-Länder-Arbeitsgruppe „Surveillance“, Kongress des Bundesverbandes der Ärztinnen und Ärzte des Öffentlichen Gesundheitsdienstes e. V. (BVÖGD-Kongress 2024) [20]).

Die vorliegende Studie ist von Relevanz, da sie erstmals die während der Pandemie zur Unterstützung des ÖGD geleisteten RKI-Einsätze strukturiert untersucht, das Feedback der Unterstützten anonym erhebt und so dabei helfen kann, die Vorbereitung auf zukünftige Ereignisse zu verbessern. Die COVID-19-Pandemie hat gezeigt, wie wichtig eine schnelle und koordinierte Reaktion über alle Behörden auf Ausbrüche von Infektionskrankheiten für den Schutz der öffentlichen Gesundheit ist [21, 22]. Ausbruchsuntersuchungen spielen eine entscheidende Rolle bei der Eindämmung von Ausbrüchen und der Bewältigung von Gesundheitskrisen [11]. Die Fähigkeit eines öffentlichen Gesundheitssystems zur raschen Mobilisierung von Ressourcen und Fachwissen, auch vor Ort, wie z. B. durch Ausbruchsteams, ist hierbei ein wichtiger Bestandteil [23, 24].

Auf Ebene der Europäischen Union (EU) wird die Bedeutung von operativer Arbeit vor Ort erkannt und mit einem neuen Mandat gestärkt, das die Einrichtung einer EU-Gesundheits-Taskforce (EUHTF) vorsieht [25, 26]. Die Erfahrungen aus den RKI-Einsätzen und die Ergebnisse dieser Studie können Hinweise für den Entwicklungsprozess der EUHTF liefern. Dies gilt insbesondere vor dem Hintergrund, dass Deutschland das bevölkerungsreichste Land der EU ist und ein föderales Staatsmodell aufweist, in dem es einer differenzierten und partizipativen Entscheidungsfindung und Koordinierung bedarf, die auf dem Feedback der kommunalen und Landesebenen fußen soll.

Die hier beschriebene Studie weist einige Limitationen auf, die bei der Interpretation der Ergebnisse berücksichtigt werden müssen: 1) Eine freiwillige Online-Befragung kann zu Selbstselektion der Teilnehmenden und einem Selektionsbias führen [27]. Dies schränkt die Verallgemeinerbarkeit unserer Ergebnisse auf den gesamten ÖGD ein, da sich die teilnehmenden Behörden von denen, die nicht teilgenommen haben, in ihren Antworten unterscheiden können. 2) Aufgrund des anonymen Charakters der Befragung und der Tatsache, dass der Link zur Befragung nicht personalisiert war, muss darauf hingewiesen werden, dass die Möglichkeit von Doppelbeantwortungen durch eine Behörde nicht ausgeschlossen werden konnte. 3) Die Rücklaufquote war relativ gering, aber vergleichbar oder sogar höher als bei anderen veröffentlichten ÖGD-Online-Befragungen [9, 28]. Andererseits haben wir für 73 % (22/30) der RKI-Feldeinsätze Antworten erhalten. Die RKI-Ferneinsätze konnten nicht ausgewertet werden, da hierzu keine Antworten vorlagen.

Nicht zuletzt hätte ggf. die Ergänzung der vorliegenden Befragung durch qualitative Methoden wie Expert/-innen-Interviews oder Fokusgruppen mit Mitarbeitenden von GÄ, LB oder RKI zusätzliche Erkenntnisse liefern können sowie eine bessere Interpretation der vorliegenden Evaluationsergebnisse ermöglichen können.

## Fazit

Die Ergebnisse dieser Studie zeigen, dass die Mehrheit der Behörden mit den RKI-Einsätzen und deren Rechtzeitigkeit zufrieden war, trotz des möglichen zusätzlichen Arbeitsaufwands. Diese Einsätze waren insbesondere bei der Beantwortung epidemiologischer Fragen hilfreich. Andererseits wurden auch Wissenslücken im ÖGD zum Prozess der Amtshilfeersuchen detektiert.

Aufgrund der Ergebnisse unserer Evaluation empfehlen wir, RKI-Einsätze bei epidemiologischen Bedarfen in Betracht zu ziehen, Anforderungs- und Einsatzprozesse als Vorbereitung auf zukünftige infektionsepidemiologische Lagen weiter zu etablieren und Wissen und Informationen dazu durch z. B. Schulungen zu vermitteln. Sollten ähnliche Konzepte der schnellen Entsendung von Teams zur Unterstützung von Ausbruchsuntersuchungen auf subnationalen Ebenen umgesetzt werden, empfehlen wir auf der Grundlage unserer Ergebnisse und Erfahrungen, klare, bekannte Prozesse für die Anforderung von Einsätzen einzurichten, die Vorbesprechungen und Nachbesprechungen standardmäßig durchzuführen sowie die Einsätze mit den anfordernden Behörden abschließend zu bewerten.

## Supplementary Information

Onlinematerial 1: Fragebogen für die Bewertung der RKI-Einsätze zur Unterstützung der subnationalen Gesundheitsbehörden in Deutschland während der COVID-19-Pandemie, 2020-2021

Onlinematerial 2: Tabelle A1. Angaben zu den Einsätzen des Robert Koch-Instituts (RKI) zur Unterstützung der subnationalen Gesundheitsbehörden in Deutschland während der COVID-19-Pandemie 2020-2021 stratifiziert nach Gesundheitsämtern (GÄ) und Landesbehörden (LB).
