# Supplementary material for: Evaluation of the Robert Koch Institute’s missions for COVID-19 outbreak investigations by local and state health authorities in Germany
Source: Bundesgesundheitsblatt Gesundheitsforschung Gesundheitsschutz. 2025 Mar 19;68(4):458–66. [Article in German] doi: 10.1007/s00103-025-04032-6 (PMC11950042; doi:10.1007/s00103-025-04032-6)
Supplement: Supplementary file 1 — Onlinematerial 1: Fragebogen für die Bewertung der RKI-Einsätze zur Unterstützung der subnationalen Gesundheitsbehörden in Deutschland während der COVID-19-Pandemie, 2020-2021 [file 103_2025_4032_MOESM1_ESM.pdf]

Onlinematerial 1 zur Originalarbeit:

Bewertung der Einsätze des Robert Koch-Instituts für COVID-19-Ausbruchuntersuchungen durch subnationale Gesundheitsbehörden

Mario Martín-Sánchez [1,2], Claudia Siffczyk[1], Anna Loenenbach[1], Katja Kajikhina[1], Nadine Zeitlmann[1]

1. Abteilung für Infektionsepidemiologie, Robert Koch-Institut, Berlin, Deutschland

2. ECDC Fellowship Programme, Field Epidemiology path (EPIET), European Centre for Disease Prevention and Control (ECDC), Stockholm, Sweden

Korrespondenzadresse

Nadine Zeitlmann

Abteilung für Infektionsepidemiologie, Robert Koch-Institut

Seestr. 10, Berlin, Deutschland

ZeitlmannN@rki.de

Fragebogen für die Bewertung der RKI-Einsätze zur Unterstützung der subnationalen Gesundheitsbehörden in Deutschland während der COVID-19-Pandemie, 2020-2021.

Online-Befragung „Landing Page“

Sehr geehrte Kolleg:innen, sehr geehrte Damen und Herren,

vielen Dank, dass Sie und Ihre Behörde sich für die Online-Befragung des Robert Koch-Instituts (RKI) zur ,,Retrospektiven Untersuchung der Feldeinsätze des RKI im Rahmen der COVID-19-Pandemie‘‘ interessieren.

Die Befragung umfasst eine Feedbackerhebung zu Amtshilfeersuchen gemäß § 4 IfSG und den verschiedenen Einsätzen des RKI in den Jahren 2020 bis 2021 im Rahmen der COVID-19-Pandemie, in welchen Gesundheitsämter (GÄ) und Landesbehörden (LB) bei der Bewältigung von Aufgaben und Ausbrüchen unterstützt wurden.

Die Befragung richtet sich an ALLE Gesundheitsämter (GÄ) und Landesbehörden (LB) – auch die, die kein Amtshilfeersuchen (gemäß § 4 IfSG) im Rahmen der COVID-19-Pandemie gestellt haben.

Mit Hilfe der Online- Befragung möchten wir folgenden Themenschwerpunkten nachgehen:

- Evaluation der Wirksamkeit der RKI-Feldeinsätze für das Ausbruchsmanagement von GÄ/LB (im Sinne von u. a. Rechtzeitigkeit, Zielstellung und Ergebniserzielung)
- Identifikation potentieller Hürden und Verbesserungsmöglichkeiten der Prozesse bei Amtshilfeersuchen und Einsätzen

Relevante Aspekte zum Datenschutz der Befragung können der folgenden Seite (link) entnommen werden.

Bei weiteren Fragen zum Inhalt, Ablauf der Befragung sowie datenschutzrechtlichen Aspekten möchten wir Sie bitten, sich an das Postfach oegd-kontakt@rki.de zu wenden. Ansprechpersonen hierfür sind Nadine Zeitlmann und Claudia Siffczyk.

Vielen Dank für Ihre Unterstützung.

Das Projekt-Team

Fachgebiet 31:,,ÖGD-Kontaktstelle: Krisenmanagement, Ausbruchsuntersuchungen und Trainingsprogramme‘‘

- Nadine Zeitlmann (Projektleitung)
- Claudia Siffczyk
- Anna Loenenbach
- Dr. Katja Kajikhina
- Mario Martin Sanchez

Online-Befragung „Intro Page“

Hinweise zur Online-Befragung:

- Die Dauer der Befragung liegt zwischen 10 und 45 Minuten, je nachdem, wie viele Einsätze stattfanden.

- Es soll pro Behörde jeweils nur ein Fragebogen ausgefüllt werden. Dies kann durch die Amtsleitung (ggf. unter Zuhilfenahme von Mitarbeitenden, die zur Zeit des Einsatzes/Amtshilfeersuchens Kontakt mit dem RKI-Team hatten) geschehen, aber auch an entsprechende Mitarbeitende delegiert werden.

- Als ,,Einsatz/Unterstützung‘‘ sind im Rahmen der Befragung Einsätze eines RKI-Teams im Zeitraum Januar 2020 – September 2021 gemeint, die fachlich-epidemiologischer bzw. strategischer Natur waren und im Rahmen eines Amtshilfeersuchens stattfanden (d. h. Mitglieder des RKI-Teams wurden entweder als Feldteam für eine gewisse Zeit an die Behörde entsandt oder unterstützten als Team die Behörde virtuell).

- NICHT als ,,Einsatz/Unterstützung‘‘ sind im Rahmen der vorliegenden Studie sporadische Auskünfte/Unterstützungen des RKI oder eine Zusammenarbeit im Rahmen von Studien gemeint. Das Gleiche gilt für operative Einsätze alleinig von Containment Scouts, die einem Amtshilfeersuchen folgten.

- Ihre Behörde bleibt im Rahmen der gesamten Befragung anonym. Wir bitten deshalb darum, KEINE personenbezogenen Daten oder Namen der Behörde bzw. geografischen Angaben in den Freitextfeldern zu nennen. Auch im Rahmen der Auswertung und Darstellung der Ergebnisse bleibt diese Anonymität bestehen, d. h. auch Angaben im Freitext, die möglicherweise erkennbar einer bestimmten Behörde zuzuordnen sind, werden in der Darstellung der Ergebnisse nicht aufgeführt.

- Das Ausfüllen des Fragebogens erfolgt online mit Möglichkeit zum Zwischenspeichern. Dies bedeutet, dass man die Befragung pausieren kann und zu einem späteren Zeitpunkt durch Eingabe **einer individuellen PIN**, die durch das System bei der Unterbrechung kreiert wird, fortführen (**bitte diese PIN in diesem Fall vor dem Unterbrechen der Befragung notieren**).

Fragebogen

TEIL 1: Angaben zur Behörde und zum Amtshilfeersuchen

A. Angaben zur Behörde

Art der Behörde

1. Für welche Art von Behörde antworten Sie (Landesbehörde/GA)

Größe

   a. Falls GA: Wie groß ist die Bevölkerung des Ihrer Behörde zugehörigen Kreises? (Einfachauswahl: <100.000 Personen, 100.000-200.000 Personen, 200.000-300.000 Personen, 300.000-400.000 Personen, 400.000-500.000 Personen, >500.000 Personen, keine Angabe)

2. Wie groß war der für Infektionsschutz und Ausbruchsuntersuchung zuständige Fachbereich in Ihrer Behörde vor der Pandemie in Voll-/ und Halbzeitstellen von Mitarbeitenden (*numerische Antwort mit einer Dezimale;* keine Angabe)

3. Wie viele zusätzliche Mitarbeitende waren gleichzeitig zu Zeiten der höchsten Arbeitsbelastung für die Arbeit an COVID-19 noch hinzugezogen (ungefähre Angabe; zählen Sie sowohl externe Unterstützung z.B. Bundeswehr, Containment Scouts, von anderen Institutionen als auch interne Unterstützung z.B. Abordnungen, Zuständigkeitsübernahmen von anderen Fachbereichen mit) (Einfachauswahl: keine(r), 1-10, 11-50, 51-100, 101-200, >200, weiß nicht, keine Angabe)

Erfahrung

4. Welche Art von Ausbildungen und Erfahrungen liegen in Ihrer Behörde unter den Mitarbeitenden des für Infektionsschutz und Ausbruchsuntersuchung zuständigen Fachbereichs vor? (Mehrfachantwort; Auswahlmenü: Epikurs, Trainingsprogramme für Feldepidemiologie [z.B. EPIET, EUPHEM, PAE], langjährige praktische Erfahrungen in Ausbruchsuntersuchungen, Facharztausbildung für Öffentliches Gesundheitswesen, Lehrgänge für Amtsärzt:innen, Hygienekontrolleursausbildung /Gesundheitsaufseher:in, weitere relevante Schulungen/Lehrgänge [Freitext], keine Angabe)

B. Angaben zum Amtshilfeersuchen

5. Wurde das RKI von Ihrer Behörde im Zeitraum Januar 2020 – September 2021 bzgl. COVID-19 um Unterstützung durch ein Amtshilfeersuchen gebeten? (Einfachauswahl: Nein, „Ja, aber ausschließlich zur Anfrage von RKI-Containment Scouts“, „Ja, für den Einsatz eines RKI-Feldteams“, keine Angabe)
   a. **Falls Nein**: Was waren Gründe, dafür, dass kein Amtshilfeersuchen gestellt wurde? (Mehrfachantwort: kein Bedarf bzw. die Beratung des RKI war auch ohne Amtshilfeersuchen ausreichend um die Situation zu bewältigen, Vorgang unklar/kein Wissen zum zugehörigen Vorgang, keine Erfahrung damit, schlechte Erfahrungen mit dem RKI/Unterstützung durch andere Behörden, keine adäquate Zielstellung für den Einsatz, Befürchtung, der Aufwand des Feldeinsatzes für die Behörde und der Nutzen entsprechen sich nicht, Keine Ressourcen, Sonstige Hürden (Freitext), Sonstiges, keine Angabe

   b. **Falls „Ja, aber ausschließlich zur Anfrage von RKI-Containment Scouts“:** Was waren Gründe, dafür, dass kein Amtshilfeersuchen für den Einsatz eines RKI-Feldteams gestellt

wurde? (Mehrfachantwort: kein Bedarf bzw. die Beratung/Unterstützung des RKI [inkl. Containment Scouts] war ausreichend um die Situation zu bewältigen, Vorgang unklar/kein Wissen zum zugehörigen Vorgang, keine Erfahrung damit, schlechte Erfahrungen mit dem RKI/Unterstützung durch andere Behörden, keine adäquate Zielstellung für den Einsatz, Befürchtung, der Aufwand des Feldeinsatzes für die Behörde und der Nutzen entsprechen sich nicht, Keine Ressourcen, Sonstige Hürden (Freitext), Sonstiges, keine Angabe)

i. Falls was anderes als kein Bedarf / Beratung des RKI ausreichend / keine Angabe angegeben wurde: Was würde Ihnen erleichtern Unterstützung durch andere Behörden/das RKI über Amtshilfe anzufragen? (*Freitext*; weiß nicht, keine Angabe)

ii. Falls Sonstiges: Spezifikation (*Freitext*)

c. **Falls „Ja, für RKI-Containment Scouts" / Nein / keine Angabe**: Glauben Sie, dass Ihre Behörde in Zukunft Interesse daran haben könnte bei Bedarf, den Einsatz eines RKI-Feldteams anzufragen (über ein Amtshilfeersuchen)? (Einfachauswahl: sehr wahrscheinlich, wahrscheinlich, unwahrscheinlich, sehr unwahrscheinlich, keine Angabe)

i. Falls sehr unwahrscheinlich oder unwahrscheinlich: Welche Gründe sprechen dagegen ein Amtshilfeersuchen anzufragen (*Freitext*; keine Angabe)

d. **Falls „Ja, für RKI-Containment Scouts"/Nein/keine Angabe:** Sonstige Mitteilungen/Weitere Kommentare (*Freitext*) → **ENDE FRAGEBOGEN**

e. **Falls „Ja; für den Einsatz eines RKI-Feldteams"**: Gab es Hürden bei der Stellung des Amtshilfeersuchens? (Einfachauswahl: Ja, Nein, weiß nicht, keine Angabe)

i. Falls Ja: Welche Hürden? (*Freitext*)

f. **Falls „Ja; für den Einsatz eines RKI-Feldteams"**: Wie oft wurde ein Amtshilfeersuchen gestellt? (Einfachauswahl: einmal, mehrmals, keine Angabe)

i. Falls keine Angabe: Sonstige Mitteilungen/Weitere Kommentare (Freitext) → **ENDE FRAGEBOGEN**

ii. Falls einmal: Fand ein Einsatz eines RKI-Feldteams statt? (Einfachauswahl: Ja, Nein, keine Angabe)

A. Falls Nein: Gründe, aus denen der Einsatz nicht stattgefunden hat (*Freitext*)

B. Falls Nein / keine Angabe: Sonstige Mitteilungen/Weitere Kommentare (*Freitext*) →**ENDE FRAGEBOGEN**

C. Falls Ja → **TEIL 2**

iii. Falls mehrmals: Anzahl (*numerische Antwort*)

iv. Falls mehrmals: Fand bei jedem Amtshilfeersuchen ein Einsatz eines RKI-Feldteams statt? (Einfachauswahl: [1] es fand nach keinem Amtshilfeersuchen ein Einsatz statt, [2] es fand nicht immer ein Einsatz statt, [3] es fand nach jedem Amtshilfeersuchen ein Einsatz statt, [4] keine Angabe)

a) Falls [1]: Gründe, dass keine Einsätze stattfanden? (*Freitext*)

b) Falls [1]/[4]: Sonstige Mitteilungen/Weitere Kommentare (*Freitext*) → **ENDE FRAGEBOGEN**

c) Falls [2]: Gründe, dass manche Einsätze nicht stattfanden (*Freitext*)

d) Falls [2]: Bei wie vielen Amtshilfeersuchen kam es zu einem Einsatz? (Anzahl) → **TEIL 2** (*für jeden Einsatz separat ausgefüllt*

e) Falls [3] → **TEIL 2** (*für jeden Einsatz separat ausgefüllt)*)

TEIL 2: Angaben zu den Einsätzen

[Abschnitt so oft ausgefüllt, wie Einsätze in der Behörde stattgefunden haben]

A Angaben zum Einsatz des RKI-Feldteams

6. Beginn des Einsatzes (Auswahlmenü: Monat, keine Angabe; Auswahlmenü: Jahr 2020, Jahr 2021, keine Angabe)
7. Wie viele Personen und aus welchen Behörden waren aktiv ins Ausbruchsgeschehen eingebunden?
    a. von der eigenen Behörde
        i. Einfachauswahl: Ja, Nein, keine Angabe
        ii. Anzahl: *numerische Antwort*, Anzahl unbekannt
    b. RKI-Feldteam
        i. Einfachauswahl: Ja, Nein, keine Angabe
        ii. Anzahl: *numerische Antwort*, Anzahl unbekannt
    c. Landesamt
        i. Einfachauswahl: Ja, Nein, keine Angabe
        ii. Anzahl: *numerische Antwort*, Anzahl unbekannt
    d. Landesministerium
        i. Einfachauswahl: Ja, Nein, keine Angabe
        ii. Anzahl: *numerische Antwort*, Anzahl unbekannt
    e. andere Behörden/Organisationen
        i. Name der Organisation/Behörde: *Freitext*, keine Angabe
        ii. Einfachauswahl: Ja, Nein, keine Angabe
        iii. Anzahl: *numerische Antwort*, Anzahl unbekannt

8. Waren die Mitglieder des RKI-Feldteams gleichzeitig oder in verschieden Teams nacheinander vor Ort (Einfachauswahl: gleichzeitig, nacheinander, weiß nicht, keine Angabe)
    a. **Falls nacheinander:** Wie viele RKI-teams gab es? (*numerische Antwort* (*Range: 1-10),* weiß nicht, keine Angabe)

9. Dauer des RKI-Einsatzes (in Tagen) (*numerische Antwort*, keine Angabe)

10. Art des RKI-Einsatzes (Einfachauswahl: nur virtuell, virtuell und vor Ort, nur vor Ort, keine Angabe)

11. Ausbruchssetting (Mehrfachantworten: Alten-/Pflegeheim, Arbeitsplatz/Betrieb, Gemeinschaftseinrichtung, medizinische Einrichtung, Freizeiteinrichtung, Gemeinschaftsunterkunft für Asylsuchende, Reiseunterkunft (z.B. Hotel, Kreuzfahrtschiff), Verkehrsmittel, privater Haushalt, Sonstiges, Keine Angabe)
    a. **Falls Sonstiges**: Spezifikation (*Freitext*)

12. Was war die Motivation/Anlass für die Unterstützungsanfrage beim RKI? (Mehrfachantworten: fehlende personelle Ressourcen für Maßnahmen, politischer und/oder öffentlicher Druck, zusätzliche fachliche Expertise benötigt, Sonstiges, weiß nicht, Keine Angabe)
    a. **Falls Sonstige:** Spezifikation (*Freitext*)

13. Welche Zielstellung verfolgte der RKI-Einsatz? (Mehrfachantworten: operative Unterstützung (z.B. bei der Durchführung von Maßnahmen), strategische Unterstützung [z.B. bei der Maßnahmenplanung, Krisenmanagement], fachlich-epidemiologische Fragestellung [z.B. Datenauswertungen, Hypothesentestung], Sonstige, weiß nicht, Keine Angabe)

a. **Falls Sonstige:** Spezifikation (*Freitext*)

B Vor dem Einsatz

14. Wurde(n) vor dem Einsatz:

    a. …konkrete Ziele der Ausbruchsuntersuchung festgelegt? (Einfachauswahl: Ja, Nein, weiß nicht, keine Angabe)
    b. …Erwartungen an das RKI-Team festgelegt? (Einfachauswahl: Ja, Nein, weiß nicht, keine Angabe)
    c. …sich auf konkrete erwartete Ergebnisse/Outputs aus dem Einsatz geeinigt (Einfachauswahl: Ja, Nein, weiß nicht, keine Angabe)
    d. …eine Vorbesprechung mit dem RKI durchgeführt (Einfachauswahl: Ja, Nein, weiß nicht, keine Angabe)
        i. Falls Ja, welche Behörden waren in die Vorbesprechung involviert? (Mehrfachantworten: Landesamt, Landesministerium, Gesundheitsamt, Weitere, Weiß nicht, Keine Angabe)

15. Verbesserungsvorschläge für den Entsendungsprozess (*Freitext*, weiß nicht, keine Angabe)

C Während des Einsatzes

16. Inwieweit stimmen Sie folgenden Aussagen zu (Einfachauswahl [pro Aussage]: stimme überhaupt nicht zu, stimme nicht zu, stimme zu, stimme völlig zu; nichtzutreffend, keine Angabe):

    Während des Einsatzes…
    a. …das RKI war nach dem Amtshilfeersuchen schnell genug vor Ort
    b. …konnte die damalige Arbeitslast durch den Einsatz verringert werden
    c. …haben die Mitarbeitenden des RKI im Management des Ausbruchs als helfende Hände wirksam unterstützt
    d. …konnten (neue) Infektionsschutzmaßnahmen durchgeführt bzw. bestehende angepasst werden
    e. …wurde politischer /öffentlicher Druck verringert
    f. …wurden Arbeitsabläufe verbessert /optimiert

17. In welchen Bereichen und zu welchem Ausmaß führte der Einsatz des RKI an Ihrer Behörde zu Mehrarbeit? (Einfachauswahl [pro Unterpunkt]: keine Mehrarbeit, angemessene Mehrarbeit, unverhältnismäßig hohe Mehrarbeit, keine Angabe)

    a. … durch Reiseorganisation (Anreise/Unterkunft etc.)
    b. …Bereitstellung von Arbeitsplatz/ Technik / Zugangsberechtigungen
    c. …Einarbeitung/Erklärung der Arbeitsprozesse
    d. …Abstimmungsprozesse
    e. …Mehrarbeit bei Datenerhebung
    f. …Sonstiges
        i. Falls Sonstiges: Spezifikation (*Freitext*)

18. War die Dauer des Einsatzes den Ergebnissen angemessen? (Einfachauswahl; Ja, „Nein, zu kurz“, „Nein, zu lang“, weiß nicht, keine Angabe)

D Nach dem Einsatz (Ergebnisse)

19. Wurden die erwarteten Ergebnisse/Outputs durch den Einsatz erreicht (z.B. Analysen,

Veröffentlichungen, Berichte, ...)? (Einfachauswahl: Ja, „Ja, aber nicht rechtzeitig“, Nein, der Einsatz hatte nicht zum Ziel konkrete Outputs zu liefern, keine Angabe)

20. Inwieweit stimmen Sie folgenden Aussagen zu (Einfachauswahl [pro Aussage]: stimme überhaupt nicht zu, stimme nicht zu, stimme zu, stimme völlig zu; nichtzutreffend, keine Angabe):

    Durch den Einsatz wurde(n)…
    a. …die Dauer der Ausbruchsuntersuchung verringert (Untersuchung wurde schneller abgeschlossen),
    b. …notwendige Erkenntnisse gewonnen / wichtige epidemiologische Fragestellungen beantwortet
    c. …neues Wissen/Expertise durch die RKI-Mitarbeitenden vermittelt
    d. …Ergebnisse der Ausbruchsuntersuchung wissenschaftlich kommuniziert (z.B. Publikationen)
    e. …Eine gute Dokumentation des Ausbruchsgeschehens erreicht
    f. …Sonstiges (Freitext)
        i. Falls Sonstiges: Spezifikation (*Freitext*)

21. Wie zufrieden waren Sie/Ihre Behörde mit folgenden Aspekten: (Einfachauswahl [pro Aspekt]: überhaupt nicht zufrieden, nicht zufrieden, zufrieden, sehr zufrieden,keine Angabe):

    a. Erreichbarkeit der Ansprechpersonen am RKI
    b. Die Kommunikation mit dem RKI-Feldteam
    c. Die weitere Zusammenarbeit mit dem RKI-Feldteam
    d. Der Einsatz des RKI-Feldteams insgesamt

22. Was kann das RKI zukünftig im Bereich Ausbruchsunterstützung besser machen? (*Freitext,* keine Angabe)

23. Seit dem Einsatz werden die gewonnenen Erkenntnisse / geänderten Arbeitsweisen / Vorgehensweisen bzw. Auswertungen weiter (gelegentlich oder routinemäßig) umgesetzt (Einfachauswahl: stimme überhaupt nicht zu, stimme nicht zu, stimme zu, stimme völlig zu; nichtzutreffend, keine Angabe)

24. Bitte vervollständigen Sie den Satz: Durch den RKI-Einsatz ist unsere Behörde nun eher in der Lage… (*Freitext*, weiß nicht, keine Angabe) → **TEIL 3** (wenn die Informationen für den letzten Einsatz beantwortet wurden)

TEIL 2: Zukünftige Unterstützung

25. Würden Sie/Ihre Behörde das RKI bei Bedarf erneut im Rahmen eines Amtshilfeersuchens für einen Einsatz eines RKI-Feldteams kontaktieren? (Einfachauswahl: sehr wahrscheinlich, wahrscheinlich, unwahrscheinlich, sehr unwahrscheinlich, keine Angabe)

26. Inwieweit würden Sie anderen Gesundheitsämtern und Landesbehörden empfehlen ein RKI-Feldteam über Amtshilfe einzuladen? (Einfachauswahl: sehr wahrscheinlich, wahrscheinlich, unwahrscheinlich, sehr unwahrscheinlich, keine Angabe)

   a. Falls unwahrscheinlich/sehr unwahrscheinlich: Gründe warum sie dies wahrscheinlich nicht empfehlen würden? (*Freitext*, keine Angabe)

27. Sonstige Mitteilungen/Weitere Kommentare (*Freitext*, keine Angabe) → **ENDE FRAGEBOGEN**
