# Supplementary material for: Evaluation of the Robert Koch Institute’s missions for COVID-19 outbreak investigations by local and state health authorities in Germany
Source: Bundesgesundheitsblatt Gesundheitsforschung Gesundheitsschutz. 2025 Mar 19;68(4):458–66. [Article in German] doi: 10.1007/s00103-025-04032-6 (PMC11950042; doi:10.1007/s00103-025-04032-6)
Supplement: Supplementary file 2 — Onlinematerial 2: Tabelle A1. Angaben zu den Einsätzen des Robert Koch-Instituts (RKI) zur Unterstützung der subnationalen Gesundheitsbehörden in Deutschland während der COVID-19-Pandemie 2020-2021 stratifiziert nach Gesundheitsämtern (GÄ) und Landesbehörden (LB). [file 103_2025_4032_MOESM2_ESM.pdf]

*Onlinematerial 2 zur Originalarbeit:*

# Bewertung der Einsätze des Robert Koch-Instituts für COVID-19-Ausbruchuntersuchungen durch subnationale Gesundheitsbehörden

Mario Martín-Sánchez [1,2], Claudia Siffczyk[1], Anna Loenenbach[1], Katja Kajikhina[1], Nadine Zeitlmann[1]

1. Abteilung für Infektionsepidemiologie, Robert Koch-Institut, Berlin, Deutschland

2. ECDC Fellowship Programme, Field Epidemiology path (EPIET), European Centre for Disease Prevention and Control (ECDC), Stockholm, Sweden

Korrespondenzadresse

Nadine Zeitlmann

Abteilung für Infektionsepidemiologie, Robert Koch-Institut

Seestr. 10, Berlin, Deutschland

ZeitlmannN@rki.de

**Tabelle A1.** Angaben zu den Einsätzen des Robert Koch-Instituts (RKI) zur Unterstützung der subnationalen Gesundheitsbehörden in Deutschland während der COVID-19-Pandemie 2020-2021 stratifiziert nach Gesundheitsämtern (GÄ) und Landesbehörden (LB).

| | | Antworten der GÄ (14 Einsätze) | Antworten der LB (8 Einsätze) |
|---|---|---|---|
| *Variablen mit nur einer möglichen Antwort* | | *(n)* | *(n)* |
| **Beschreibung der Einsatz** | | | |
| Monat des Beginns des Einsatzes | Januar-Juni 2020 | 6 | 3 |
| | Juli-Dezember 2020 | 1 | 1 |
| | Januar-Juni 2021 | 5 | 4 |
| | Juli-September 2021 | 2 | 0 |
| Dauer des Einsatzes | ≤ 5 Tage | 6 | 8 |
| | 6-10 Tage | 1 | 0 |
| | >10 Tage | 4 | 0 |
| | Keine Angabe | 3 | 0 |
| Größe des RKI-Feldteams | 2 Personen | 3 | 1 |
| | 3 Personen | 4 | 2 |
| | 4 Personen | 4 | 4 |
| | 5 Personen | 0 | 1 |
| | Keine Angabe | 3 | 0 |
| *Variablen mit Mehrfachantwortmöglichkeit* | | | |
| **Ausbruchssettings*** | | | |
| Alten-/Pflegeheime | | 8 | 4 |
| Privater Haushalt | | 4 | 1 |
| Arbeitsplatz/Betrieb | | 4 | 1 |
| Medizinische Einrichtung | | 4 | 0 |
| Andere Ausbruchssettings | | 7 | 2 |
| **Motivation der Einsatzanfrage*** | | | |
| Zusätzliche fachliche Expertise benötigt | | 11 | 7 |
| Fehlende personelle Ressourcen für Maßnahmen | | 7 | 6 |
| Politischer und/oder öffentlicher Druck | | 4 | 8 |
| Sonstige | | 1 | 0 |
| **Einsatzvorbereitung*** | | | |
| Die Ziele des Einsatzes wurden im Voraus festgelegt | | 10 | 8 |
| Die Erwartungen an das RKI-Team wurden festgelegt | | 10 | 8 |
| Konkrete Ergebnisse/Outputs wurden vereinbart | | 7 | 5 |
| Eine Vorbesprechung mit dem RKI wurde durchgeführt | | 8 | 7 |

* Mehrfachantworten möglich, Anteil der "Ja"-Antworten.
